# Measurement of the $^{181}$Ta($n,\gamma$) cross sections up to stellar s-process temperatures at the CSNS Back-n

**DOI:** 10.1038/s41598-023-39603-7

**Published:** 2023-08-04

**Authors:** Zhendong An, Weiwei Qiu, Wei Jiang, Gaole Yang, Xiankai Li, Zhengfa Liao, Ziyue Zhuang, Xiaoping Zhang, Shengli Chen, Chenchen Guo, Erxi Xiao, Xiao Fang, Xinxiang Li, Hongwei Wang, Xinrong Hu, Bing Jiang, Wenqing Shen, Jincheng Wang, Jie Ren, Xichao Ruan, Dexin Wang, Su-Yalatu Zhang, Wen Luo, Zhichao Zhu, Haoyang Lan, Zongwei Cao, Xu Ma, Yingdu Liu, Pusen Wang, Yi Yang, Ping Su, Xiangai Deng, Wanbing He, Yugang Ma, Chunwang Ma, Yuting Wang, Pengqin He, Renguang Tang, Tao Zhou, Jing Wang, Han Yi, Yue Zhang, Yonghao Chen, Ruirui Fan, Keqing Gao, Qiang Li, Kang Sun, Zhixin Tan, Minhao Gu, Hantao Jing, Jingyu Tang

**Affiliations:** 1 School of Physics and Astronomy, Sun Yat-sen University, Zhuhai, 519082 China; 2 Sino-French Institute of Nuclear Engineering and Technology, Sun Yat-sen University, Zhuhai, 519082 China; 3 State Key Laboratory of Lunar and Planetary Sciences, Macau University of Science and Technology, Macau, 999078 China; 4 CNSA Macau Center for Space Exploration and Science, Macau, 999078 China; 5 Shanghai Institute of Applied Physics, Chinese Academy of Sciences, Shanghai, 201800 China; 6 Institute of High Energy Physics, Chinese Academy of Sciences, Beijing, 100049 China; 7 Spallation Neutron Source Science Center, Dongguan, 523803 China; 8 School of Nuclear Science and Technology, University of South China, Hengyang, 421001 China; 9 University of Chinese Academy of Sciences, Beijing, 100049 China; 10 Shanghai Advanced Research Institute, Chinese Academy of Sciences, Shanghai, 201210 China; 11 Key Laboratory of Nuclear Data, China Institute of Atomic Energy, Beijing, 102413 China; 12 College of Mathematics and Physics, Inner Mongolia Minzu University, Tongliao, 028000 China; 13 Institute of Nuclear Physics, Inner Mongolia Minzu University, Tongliao, 028000 China; 14 Shool of Materials Science and Engineering, Xiangtan University, Xiangtan, 411100 China; 15 Key Laboratory of Nuclear Physics and Ion-beam Application (MOE), Institute of Modern Physics, Department of Nuclear Science and Technology, Fudan University, Shanghai, 200433 China; 16 Institute of Particle and Nuclear Physics, Henan Normal University, Xinxiang, 453007 China; 17 School of Physics, Henan Normal University, Xinxiang, 453007 China; 18 School of Nuclear Science and Technology, University of Science and Technology of China, Hefei, 230027 China

**Keywords:** Astronomy and astrophysics, Nuclear physics, Geochemistry

## Abstract

The neutron capture cross section of $^{181}$Ta is relevant to *s*-process of nuclear astrophysics, extraterrestrial samples analysis in planetary geology and new generation nuclear energy system design. The $^{181}$Ta($n,\gamma$) cross section had been measured between 1 eV and 800 keV at the back-streaming white neutron facility (Back-n) of China spallation neutron source(CSNS) using the time-of-flight (TOF) technique and $\hbox {C}_{6}\,\hbox {D}_{6}$ liquid scintillator detectors. The experimental results are compared with the data of several evaluated libraries and previous experiments in the resolved and unresolved resonance region. Resonance parameters are extracted using the R-Matrix code SAMMY in the 1–700 eV region. The astrophysical Maxwell average cross section(MACS) from *kT* = 5 to 100 keV is calculated over a sufficiently wide range of neutron energies. For the characteristic thermal energy of an astrophysical site, at *kT* = 30keV the MACS value of $^{181}$Ta is 834 ± 75 mb, which shows an obvious discrepancy with the Karlsruhe Astrophysical Database of Nucleosynthesis in Stars (KADoNiS) recommended value 766 ± 15 mb. The new measurements strongly constrain the MACS of $^{181}$Ta($n,\gamma$) reaction in the stellar s-process temperatures.

## Introduction

**Figure 1:**
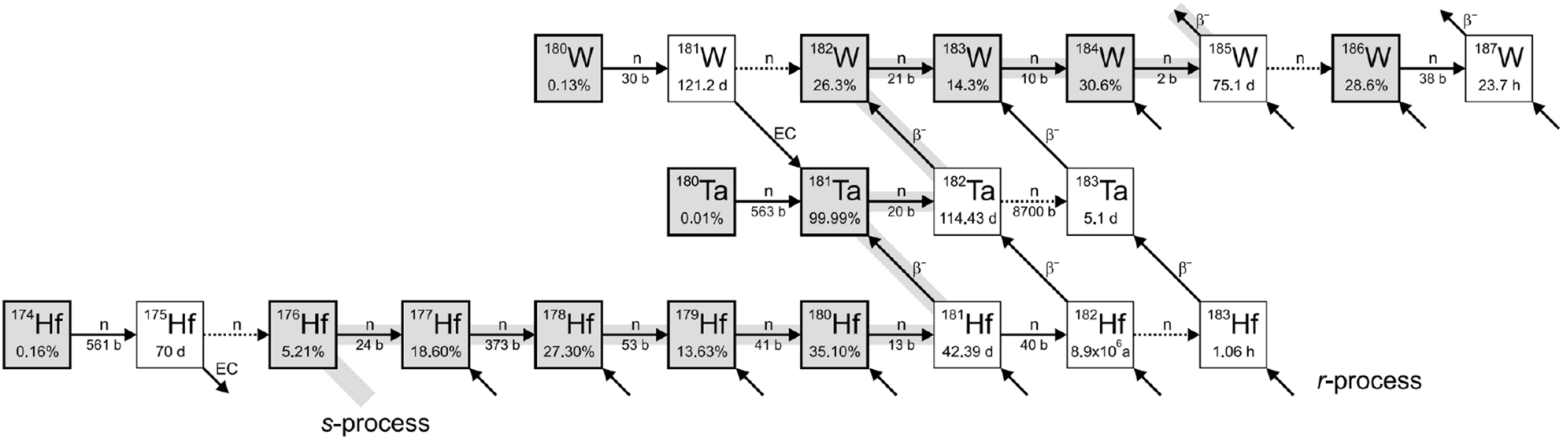
The neutron capture path of the *s*-process along the $\beta$-stability valley.

Most of the elements heavier than iron in the universe are primarily synthesized by two neutron capture processes in stars, i.e., the slow neutron capture process (*s*-process)^1^ and the rapid neutron capture process (*r*-process)^2^. The neutron capture time scale of the *s*-process is of the order of a year, which is much slower than typical $\beta$ decay times^2^. Hence, the *s*-process is mainly along the $\beta$ stability valley as indicated in Fig. 1 and contributes about half of the elemental abundances between Fe and Bi^1^. In contrast, neutron capture in the *r*-process occurs on a time scale of milliseconds, which is much faster than $\beta$ decays^2,3^. Therefore, the *r*-process ends only when it approaches the neutron drip line, which finally forms stable neutron-rich nuclei (*r*-nuclei) through a series of $\beta$-decays^2^. The *r*-process produces about half of the heavy elements found in nature^4^.

**Figure 2:**
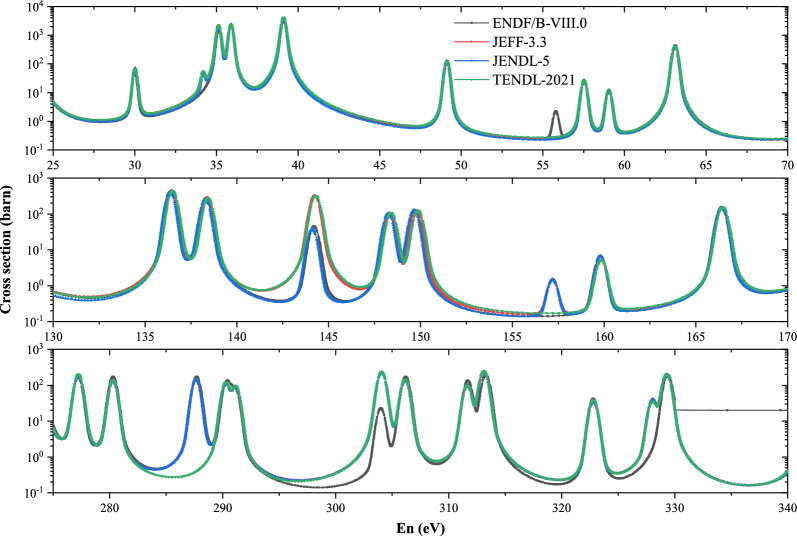
The differences of four evaluated library: ENDF/B-VIII.0, JENDL-5, JEFF-3.3 ,TENDL-2021 and JENDL-5.

Natural tantalum has two stable isotopes, the stable isotope ${}^{181}$Ta (99.988%) and the long-lived isotope ${}^{180}$Ta (0.012%), which has a half-life of $7.15\times 10^{15}$ years. $^{180}$Ta is produced by two minor branchings in the *s*-process along the stable hafnium isotopes which is discussed by Kappeler et al.^5^ and Malatji et al.^6^. $^{181}$Ta is produced by the s-process, its ($n,\gamma$) cross sections and MACS at 30 keV are of great significance in nuclear astrophysics for understanding the reaction path of the *s*-process^7,8^. However, according to the EXFOR library, high-precision, continuous measurements of capture cross sections in the resolved resonance region are not sufficient. Comparisons of the evaluated library ENDF/B-VIII.0^9^, JEFF-3.3^10^, TENDL-2021^11^ and JENDL-5^12^ also exhibit notable discrepancies in ($n,\gamma$) cross sections for $^{181}$Ta($n,\gamma$) at these energies in Fig. 2. There is a lot of experimental MACS at *kT* = 30 keV, however, different equipment and measurement methods make the experimental results vary greatly.

The moon was formed by a violent, head-on collision between the early Earth and a “planetary embryo” called Theia approximately 100 million years after the Earth formed. As one of the short-lived radioactive systems, the extinct $$ ^{182}$$Hf-$$ ^{182}$$W system is a versatile tool for investigating potential isotopic differences between the Earth and Moon, which provide critical constraints on the formation and evolution of terrestrial planets^13–15^. $$^{182}$$W isotope studies on lunar and asteroids samples should pay attention to the effects of cosmic rays particularly. The extraterrestrial samples exposed to cosmic rays will undergo a $$ ^{181}$$Ta($$n,\gamma $$)$$ ^{182}$$Ta($$\beta ^-$$)$$ ^{182}$$W reaction, which cause the measured value of $$^{182}$$W is too high compared to the actual value. How to quantitatively correct the isotope effect caused by the radiation process of cosmic rays is a major problem for the high-precision isotope analysis of lunar and asteroids samples^16^.

In addition, natural tantalum has high melting point, good mechanical properties at low and high temperatures, and good corrosion resistance^17^. Tantalum and its alloys may be used as a reactivity control and refractory material in fast reactors, space reactors^18^ and fusion reactors^19–21^. Many scholars have done a lot of research on this. Therefore, the high-precision measurement of the $^{181}$Ta ($n,\gamma$) cross section will be beneficial to predict the behavior of tantalum in the reactor.

This work provides $^{181}$Ta ($n,\gamma$) cross sections data up to energies of 700 eV in the resolved resonance region. In combination with average neutron capture cross sections determined for neutron energies up to 800 keV in the unresolved resonance region, we also calculated Maxwellian averaged cross sections for the entire range of astrophysical interest. And we present the comparative analysis results between our experimental data and the evaluated database or the existing experimental data, including the comparison of neutron capture cross-section and the MACS at kT = 5–100keV. The new measurements strongly constrain the MACS of $^{181}$Ta($n,\gamma$) reaction in the stellar s-process temperatures.

## Experimental setup and methods

### Back-n at China spallation neutron source

**Figure 3:**
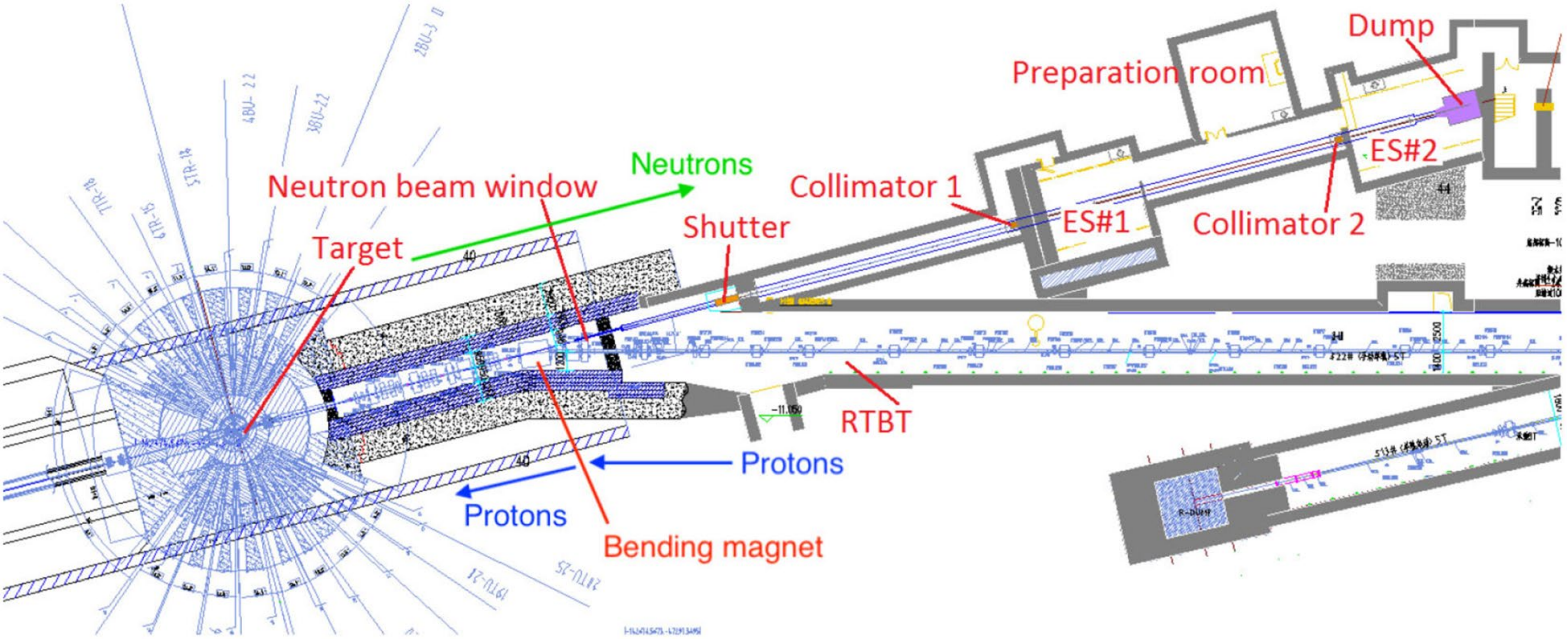
Layout of the Back-n beam line at CSNS.

CSNS is a large scientific facility in Dongguan, China, built in early 2018^22^. With the first phase (CSNS-I) of 100 kW in beam power, the accelerator can provide protons with energy of 1.6 GeV at 25 Hz pulse repetition rate to bombard a spallation target made of tungsten^23^. CSNS adds a 15° deflection magnet on the proton beam line to separate the neutron beam (back-streaming neutron, Back-n in Fig. 3) which is flowing back from the proton beam incident channel. Earlier studies show that the back-streaming neutrons from the spallation target has an excellent energy spectrum from thermal to several hundred MeV, which makes it suitable to be exploited as a white neutron source for nuclear data measurements^24,25^.

The main objectives of the experimental activity of Back-n facility are nuclear data measurement, basic nuclear physics, particle physics, neutron radiation effect and neutron photography. In the field of nuclear data measurement, Back-n focus on the accurate measurements of neutron cross sections related to nuclear astrophysics, and the collection of nuclear data related to emerging nuclear technologies for energy production, for example, thorium based molten salt reactor, R&D of accelerator-driven systems (ADS) and nuclear-waste transmutation.

Back-n at CSNS has two experimental endstations: the endstation 1 (ES#1) with a flight path of 55 m and the endstation 2 (ES#2) with a flight path of 76 m. Chen et al.^26^ measured the neutron flux of ES#2 at 7.03 $\times$ 10$^{6}$ neutrons/cm$^{2}$/s when the CSNS was operated at 100 kW in the double-bunch mode of the accelerator^23,27^. The experiment of neutron capture cross sections was carried out in ES#2 because of its lower neutron and gamma rays background compared to ES#1^28,29^. Moreover, the neutron time resolution in the ES#2 is better than that in the ES#1 for its longer neutron flight path^30^. At the end of the neutron beam line ($\sim$78 m) there is a beam dump used for reduce the backgrounds of neutrons and $\gamma$-rays.

In the neutron beam transport line, there are three neutron collimators, the neutron shutter, collimator 1 and collimator 2. By adjusting the size and shape of 3 collimators, neutron beam spot of different shapes and sizes can be obtained. In the experiment, a combination of three circular collimators of $\phi 50- \phi 15- \phi 40$ was used, and the obtained neutron beam could completely cover the sample. A detailed description of the Back-n facility and its characteristics can be found in Refs.^25^.

### C$_{6}$D$_{6}$ scintillation detector and samples

**Figure 4:**
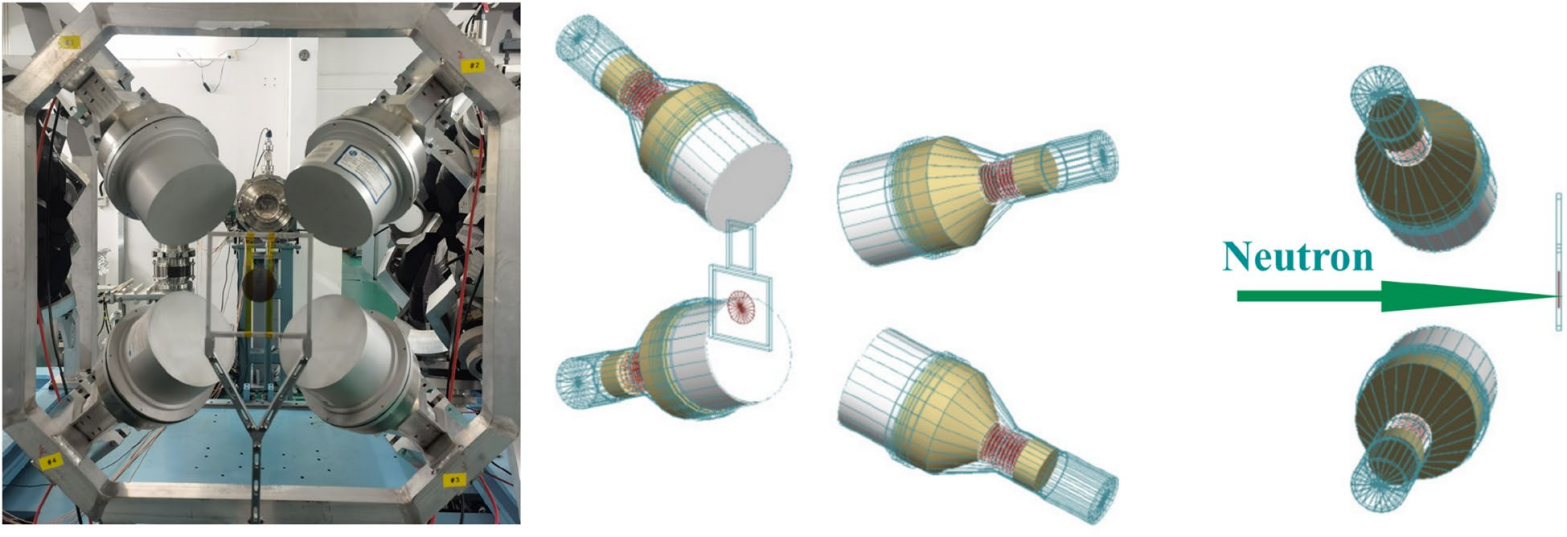
Layout of four $\hbox {C}_6\,\hbox {D}_6$ detectors in ES#2.

The prompt $\gamma$ rays detector system at the center of ES#2 consists of four $\hbox {C}_6\,\hbox {D}_6$ detectors^31^, one aluminum detector’s brackets and one aluminum sample square holder, as shown in Fig. 4. The $\hbox {C}_6\,\hbox {D}_6$ liquid scintillator is EJ315, which is produced by ELJEN Technology Corporation. The scintillator was contained in a 1.50 mm thick aluminum cell with a diameter of 130.00 mm and a length of 76.20 mm. The photomultiplier tube(PMT) coupled to the scintillator is ETEL 9390KEB, which is produced by ET Enterprises Limited.

The C${}_6$D${}_6$ detectors are placed upstream of the sample, and the detector axis is at an angle of 110${}^\circ$ relative to the neutron beam. The distance between the front center of the C${}_6$D${}_6$ detectors and the sample target center is 150 mm, while it is 80 mm between the front center of the C${}_6$D${}_6$ detectors and the neutron beam axis. A neutron conversion layer consisting of a 360 $\upmu$g/cm${}^{2}$ ${}^{6}$LiF film deposited on a 10-$\upmu$m-thick aluminum foil is placed in the neutron beam line at the front end of ES#1 and is part of a ${}^{6}$LiF–Si detector array with eight separated Si detectors.

The PMT delivered a typical anode signal with 18 ns rise time and about 80 ns decay time, which is much faster than the dynode signals, into the Back-n general-purpose data acquisition system (DAQ). The DAQ can digitize the analog signals into full waveform data with 1 GS/s sampling rate and 12 bits resolution. The time stamp of the ($n,\gamma$) signals and that of the pulsed proton beam are recorded by DAQ, so the incident neutron energy can be determined by the time-of-flight method (TOF).

Our work was carried out at experimental station ES#2. A thin foil of a cadmium absorber was placed at the front of the neutron shutter to absorb neutrons with an energy lower than 0.5 eV for avoiding the overlapping between consecutive neutron pulses. In addition, a Ag–Co filter with a total thickness of 1.0 + 1.0 mm was used to determine the in-beam $\gamma$-ray background by employing the black resonance method. For our measurements, the shutter and collimators had an inner diameter of $\Phi 50+\Phi 15+\Phi 40$ mm, resulting in a circular Gaussian-shaped beam profile with a diameter of around 40 mm at the sample position.

A total of four samples were used in the measurements: (i) the natural tantalum under study, The natural tantalum sample consisted of $^{181}$Ta with purity of 99.98%. The minor isotope of tantalum, $^{180}$Ta, is 120 ppm, which is on the same order as the concentration of other impurities in the sample. The contributions to the capture yield from both $^{180}$Ta and other impurities were thus assumed to be negligible; (ii) a ${}^{197}$Au sample for experimental setup verification and flux normalization,such as the flight distance can be calibrated and determined by $^{197}$Au standard sample; (iii) a natural carbon sample,which is used to simulate the neutron scattering and surroundings $\gamma$-rays background; and (iv) a lead sample to determine the backgrounds simulation due to the in-beam $\gamma$ rays. The $^{nat}$Ta sample was irradiated with neutrons for 17 hours in proton beam power stable at 125 kW. More details are given in Table 1.

**Table 1:** Properties of the samples used in the measurement.

Sample	Formula	Mass (g)	Diameter (mm)	Thickness (mm)	Area density(atom/barn)	Measure time (h)
$^{181}$Ta	> 99.98%	11.330 ± 0.001	40 ± 0.1	0.54 ± 0.02	5.612 $\times$ 10$^{-3}$	17
$^{nat}$C	> 99.99%	2.581 ± 0.001	40 ± 0.1	1.01 ± 0.02	1.18 $\times$ 10$^{-2}$	8
$^{nat}$Pb	> 99.99%	13.930 ± 0.001	40 ± 0.1	0.98 ± 0.02	3.220 $\times$ 10$^{-3}$	10
$^{197}$Au	> 99.99%	13.930 ± 0.001	40 ± 0.1	0.20 ± 0.02	1.174 $\times$ 10$^{-3}$	13
Empty	–	–	–	–	–	13

### Double-bunch unfolding

The CSNS proton accelerator operates in double-bunch mode. Proton beams with a time interval of 410 ns cause a superposition of event distributions. Therefore, the time resolution will be degraded without unfolding, especially in the neutron energy region higher than 500 eV. To solve this problem, we adopt the double-bunch unfolding method in Ref.^32^ to obtain better time and energy resolution. The double-bunch distribution can be treated as the superposition of two identical single-bunch distributions,

1 $$\begin{aligned} D_{i}=0.5(S_{i}+S_{i-\Delta }), \end{aligned}$$

where $D_{i}$ is the count of *ith* energy bin in the case of double-bunch mode, $S_{i}$ represents the count of *ith* energy bin in the case of single-bunch mode and $\Delta$ indicates number of energy bins corresponding to the offset of 410 ns. In this work, $D_{i}$ was obtained in experiment, what we need is the value of $S_{i}$. Based on Bayesian theorem and iterative algorithm, we can obtain the unfolding formula as follows:

2 $$\begin{aligned} S_{i}^{(k+1)}=D_{i}\frac{S_{i}^{(k)}}{S_{i}^{(k)}+S_{i-\Delta }^{(k)}} +D_{i+\Delta }\frac{S_{i}^{(k)}}{S_{i+\Delta }^{(k)}+S_{i}^{(k)}}, \end{aligned}$$

where (*k*) indicates the *kth* iteration.

The reliability and accuracy of double-bunch unfolding have been tested with simulated data and experimental data^32,33^ and meet the requirements for most of the applications at Back-n with the Bayesian unfolding method studied in more depth and probably providing higher accuracy.

### Pulse-weight weighting technique

**Figure 5:**
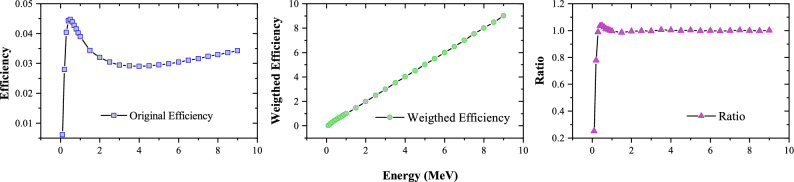
(**a**) The $\hbox {C}_6\,\hbox {D}_6$ original efficiency. (**b**) Weighted efficiency. (**c**) The ratio of weighted efficiency to $\gamma$-ray energy. For energy below 1 MeV, the weighted efficiency is not proportional to the energy, the influence of weight function failure can be eliminated by setting a threshold when processing the PH spectrum.

Due to the complex de-excitation path of the neutron capture compound nucleus, the $\hbox {C}_6\,\hbox {D}_6$ detector layouts are based on the assumption that only one de-excited $\gamma$-ray is measured in the experiment, that is, the detection efficiency is independent of the energies of cascade $\gamma$-rays^34,35^. The detection efficiency of $\hbox {C}_6\,\hbox {D}_6$ generally does not increase linearly with E$_{\gamma }$, but varies non-linearly with E$_{\gamma }$, as shown in Fig. 5. To solve this problem, Maier-Leibnitz first proposed the pulse height weighting technique (PHWT). PHWT was first applied to measure the neutron capture cross sections with C$_{6}$F$_{6}$ detector by Macklin and Gibbons^36^. PHWT demand a detailed weight function, which can be obtained by simulation using Monte Carlo method, to make the detection efficiency $\varepsilon _{\gamma }$ proportional to the $\gamma$-rays energy E$_{\gamma }$

3 $$\begin{aligned}{} & {} \varepsilon _{\gamma }=\alpha {E_\gamma }, \end{aligned}$$

4 $$\begin{aligned}{} & {} \varepsilon _c =1-\prod _{i=1}^{m}(1-\varepsilon _{\gamma i}) \simeq \sum _{i=1}^{m}\varepsilon _{\gamma i} =\alpha \sum _{i=1}^{m}E_{\gamma i} =\alpha E_c, \end{aligned}$$

Equation (4) shows that the detection efficiency of capture events is proportional to the total excitation energy of the compound nucleus. In order to achieve linear relationship between $\varepsilon _{\gamma }$ and E$_{\gamma }$, a weight function in the form of a polynomial functions is introduced, which can be expressed by

5 $$\begin{aligned} W(E_d)=\sum _{k=0}^{4}a_k E_{d}^{k}, \end{aligned}$$

where the W is the weight function, $$E_{d} $$ is the deposit energy, and $$ a_{k} $$ can be determined by least squares method:

6 $$\begin{aligned} \chi ^2=\sum [\alpha E_{\gamma j}- \int _{E_L}^{\infty }R(E_d,E_{\gamma j})W(E_d)dE_d]^2, \end{aligned}$$

where the $$ E_{\gamma j} $$ is the energy of $$ \gamma $$-rays of group *j* from the Geant4 simulation. The simulated $$ \gamma $$-rays with energy ranging from 0.1 to 9 MeV were produced from the $$ ^{nat}$$Ta sample, then emitted homogeneously and partial $$ \gamma $$-rays was detected by the $$\hbox {C}_6\,\hbox {D}_6$$ detectors with a deposit energy $$E_{d} $$. $$ R(E_d, E_{\gamma j}) $$ are counts of the pulse height (PH) spectrum with energy response function in $$ E_d $$, *E*_*L*_ is the threshold of PH spectrum. As shown in Fig. 5, we set the coefficient $$ \alpha $$ = 1. Each counts is weighed by the corresponding weight function to ensure that the $$ \varepsilon _{\gamma } $$ and $$ \gamma $$-rays energy E$$ _{\gamma } $$ fit Eq. (3). After applying the weight function to the original efficiency curve, the linear relationship between detection efficiency and energy is shown in Fig. 5, and the ratio of efficiency to energy is approximately equal to 1 for energy below 1 MeV.

Through the above processing, we can obtain the accurate weighted counts of captured events, hence the capture yield ($Y_{w}$) can be determined using the following formula:

7 $$\begin{aligned} Y_W(E)=\frac{N_w}{I \varepsilon _\gamma }=\frac{N_w}{I \alpha S_n}, \end{aligned}$$

where $N_{w}$ is the weighted pulse height spectrum count, *I* is the neutron intensity in n/cm$^{2}$/s provided by Ref.^26^, $\alpha$ = 1 /keV and $S_{n}$ is the target neutron binding energy in keV. The relationship between neutron capture yield $Y_W$ and neutron capture cross section is as follows:

8 $$\begin{aligned} Y_W(E)=(1-e^{-N_vt \sigma _t(E)})\frac{\sigma _c(E)}{\sigma _t(E)}. \end{aligned}$$

where $N_v$ is the atomic density in atom/cm$^{3}$, *t* is the target thickness in cm, $\sigma _c$ is the neutron capture cross section, and $\sigma _t$ the total cross section. Finally, we can get the formula for calculating the neutron capture cross section from the weighted counts $N_{w}$:

9 $$\begin{aligned} \sigma _c(E)=\frac{N_w}{\alpha IS_n} \times \frac{\sigma _t(E)}{1-e^{-N_v \sigma _t(E)t}}. \end{aligned}$$

### Background

**Figure 6:**
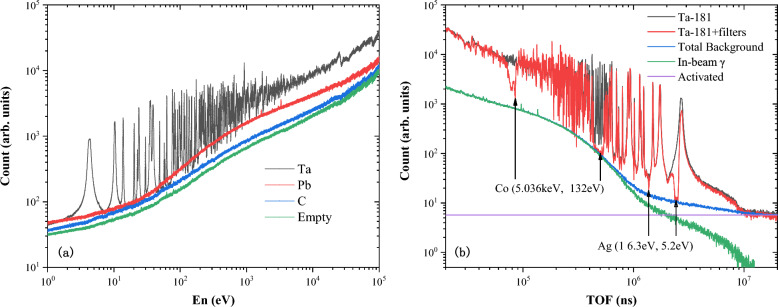
(**a**) Preprocessed and Normalized(according to the proton beam number) original spectrums of ${}^{\textbf {nat}}$Ta, ${}^{\textbf {nat}}$Pb, ${}^{\textbf {nat}}$C and empty target; (**b**) The residual TOF spectrum of the $^{181}$Ta sample with filters (red solid line), an the normalized backgrounds (blue solid line): the in-beam gamma-ray background (green triangle line), and the activation background (purple solid line).

The original spectrums preprocessed with pulse heigt weighting technique and double-bunch unfolding method are normalized by proton beam number, and shown in Fig. 6a. In order to obtain the actual counts of the tantalum neutron capture reaction, it is necessary to subtract various backgrounds, including the neutron-induced background and in-beam $\gamma$-ray background, etc. According to sample correlation, the background in our measurement of the neutron capture cross section can be divided into sample-dependent background $B_\text {sample}(t_\text {n})$ and sample-independent background $B_\text {empty}(t_\text {n})$^30^, that is

10 $$\begin{aligned} B(t_\text {n}) = B_\text {empty}(t_\text {n}) + B_\text {sample}(t_\text {n}). \end{aligned}$$

The contribution of $B_\text {empty}(t_\text {n})$ can be directly measured in the same experimental setup by keeping the sample away from the neutron beam. On the other hand, the sample-dependent background $B_\text {sample}(t_\text {n})$ is caused by interactions between the sample and all the types of in-beam particles, including the scattered-neutron-induced background $B_\text {sn}(t_\text {n})$, scattered in-beam $\gamma$-rays background $B_{\text {s}\gamma }(t_\text {n})$, and sample activation background $B_\text {ac.}$. Thus, the sample-dependent background can be expressed as^30^

11 $$\begin{aligned} B_\text {sample}(t_\text {n}) = B_\text {sn}(t_\text {n}) + B_{\text {s}\gamma }(t_\text {n})+B_\text {ac.}. \end{aligned}$$

As the cross sections of the neutron-scattering-induced and $\gamma$-ray-induced interactions vary considerably depending on the nucleus, the sample-dependent background $B_\text {sample}(t_\text {n})$ can be hardly determined through direct measurements. Thus, measurements of carbon and lead samples as well as the black resonance method were introduced to determine these backgrounds; the validity of these methods was verified through Geant4 simulations in Ref.^30^. The neutron capture cross section of carbon is considerably smaller than the scattering cross section, and the carbon scattering of $\gamma$-rays is very weak. These characteristics indicate that the carbon sample can be used to determine the scattered-neutron-induced background $B_\text {sn}(t\text {n})$ as

12 $$\begin{aligned} B_\text {sn}(t_\text {n}) = \frac{Y_\text {Ta, el}}{Y_\text {C, el}}\left( W.C_\text {C}(t_\text {n})-W.C_\text {empty}(t_\text {n})\right) , \end{aligned}$$

where $Y_\text {C, el}$ and $Y_\text {Ta, el}$ are the neutron scattering yields of the carbon and Ta targets obtained from the database.

The in-beam $\gamma$-rays originated from neutron captures in the water moderator of the spallation source. Indeed, these $\gamma$-rays can be scattered by the sample. The target and energy dependence of in-beam $\gamma$-ray background components were determined from a dedicated measurement of a lead sample and the absorption valleys of 5.18 eV, 16.3 eV, 132 eV, and 5.02 keV of the Ag–Co filter^37^, as shown in Fig. 6b. In this figure, the empty background $B_\text {empty}(t_\text {n})$ is subtracted from all spectra, and the background due to the scattered neutrons from lead sample is subtracted using Eq. (12). The figure also shows the activation background, which is determined by fitting the spectra’s platform above 11 ms ($E_\text {n}\approx 0.2$eV). In this region, neutrons are absorbed by the cadmium absorber, and the in-beam $\gamma$-rays can be ignored; the counts in the residual TOF spectrum are attributed to the activation of the sample and the surrounding materials.

**Figure 7:**
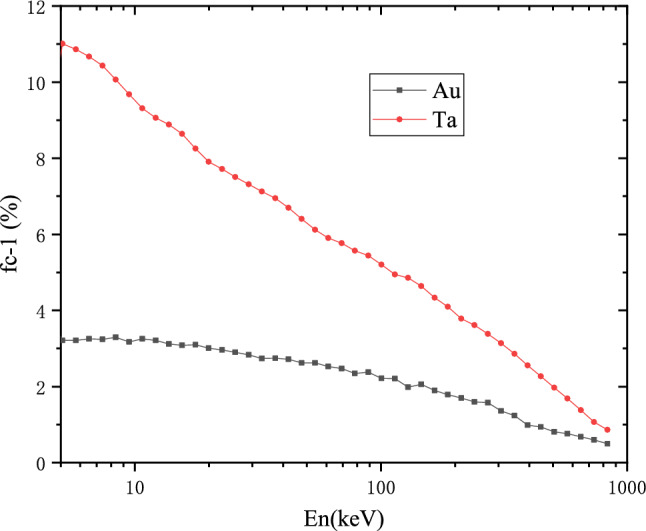
Correction factor $\textit{f}_{c}$ for $^{197}$Au and $^{181}$Ta.

### Experimental correction

In the neutron capture cross section measurement, the effect of single and multiple neutron scatterings in the capture sample is quite important in determining a capture cross section. This effect, which increases with the effective sample thickness relative to the geometrical thickness in the direction of incident neutrons, must be considered in the capture cross section measurement in which the total number of capture events in a sample is measured.

In the resolved resonance region sample-related corrections were included in the SAMMY analysis. In the unresolved region, neutron multiple scattering and self-shielding corrections in the sample have been determined with the Monte Carlo simulation. Geant4 toolkit was used to simulate the correction factor $f_{c}$ for the effect mentioned above. The results of $f_{c}$ after Geant4 toolkit simulation are shown in the Fig. 7. In this simulation, the sample size, thickness and impurities are considered to be the same as the experiment. The sample is irradiated with a parallel beam of neutrons at energy ranging from 0.3 eV to 1 MeV, and the neutron flight path in the target was recorded to get the total flight distance $F_{D}$. The correction factor $f_{c}$ refers to the ratio of $F_{D}$ to target thickness *t*

13 $$\begin{aligned} f_{c} = \frac{F_{D}}{t}. \end{aligned}$$

Therefore, Eq. (9) can be written as

14 $$\begin{aligned} \sigma _c(E)=\frac{N_w}{\alpha IS_n} \times \frac{\sigma _t(E)}{1-e^{-N_v \sigma _t(E)F_D}}. \end{aligned}$$

### Uncertainties analysis

**Table 2:** The statistic uncertainty and systematic uncertainty in this work.

Source of uncertainty	$\varepsilon$	Meaning	Value (%)
Experiment conditions	$\varepsilon _{\phi 1}$	Uncertainty of neutron energy spectrum below 150 keV	$\approx$8.00
	$\varepsilon _{\phi 2}$	Uncertainty of neutron energy spectrum above 150 keV	$\approx$4.50
	$\varepsilon _{sample}$	Uncertainty of sample parameter (see Table 1)	< 0.50
	$\varepsilon _{pbp}$	Uncertainty of proton beam power	< 1.50
Data analysis	$\varepsilon _{PHWT}$	Uncertainty from PHWT	< 3.00
	$\varepsilon _{unfolding}$	Uncertainty of unfolding	< 2.00
Statistics	$\varepsilon _{Statistic}$	Statistic uncertainty	< 2.70
Error propagation	$\varepsilon _{total}$	Total uncertainty	< 9.00

In this section, the uncertainties, including statistical and systematic, will be discussed.The statistical uncertainty comes from raw counts in a energy bin of four samples and was estimated to be < 2.70%. In fact, since the raw counts will change depending on the width of energy bins and value of $(n,\gamma )$ cross sections. Wider energy bins will help to increase the counts and reduce the statistical error, for energy > 2.4 keV), but at the same time, too wide energy bins cannot exhibit the fine resonances structure. For energy below 6.00 eV, a super fine energy bin of 0.01 eV/bins was applied with statistical error < 1.00% because of the high $(n,\gamma )$ cross section around the first resonance at 4.28 eV.

The systematic uncertainty are mainly contributed by the uncertainty of experimental conditions and data analysis method. Uncertainty of Experimental conditions contain several types including uncertainty of sample parameter, neutron energy spectrum and proton beam power. According to the measurement of the experimental samples (see Table 1), the uncertainty of sample parameter is less than 3.70%. During the experiment, the uncertainty of proton beam power is 1.50%. Li et al.^27^ measured the neutron energy spectrum of Back-n ES#2 from 1 eV to 100 MeV by $^{6}$LiF-silicon detector array. The uncertainty of neutron energy spectrum was reported as $\approx$4.50% above 2 keV and $\approx$8.00% below 2 keV. Uncertainty of data analysis method are mainly caused by the PHWT method^38^ and double-bunch unfolding process^32^. Tain et al. compared the neutron widths for the 1.15 keV resonance in $^{56}$Fe between the results treated by PHWT and the result from experiment, finding that the systematic deviations of PHWT is of the order of 2.00–3.00%^38^.

Finally, according to the error propagation, the overall experimental uncertainty is less than 9.00% in Table 2. Such a high error mainly comes from the uncertainty of the neutron spectrum (< 8%). Therefore, a good neutron energy spectrum with lower uncertainty will greatly improve the accuracy of this experiment, which also puts forward higher requirements for the CSNS neutron energy spectrum measurement team.

## Result and discussion

### R-matrix fits in the resonance energy range

The neutron capture cross sections of natural tantalum target were measured and analyzed using the R-matrix SAMMY code^39^ in the resonance energy range of 1–700 eV. In the resolved resonance region (RRR), theoretical cross sections are generated using the Reich-Moore approximation to R-matrix theory (and extensions thereof). This formulation of the Reich-Moore equations has been implemented in segment XCT of the code SAMMY, and the detailed equations can be referred to the updated users’ guide for SAMMY. Sophisticated models are used to describe the experimental situation: Data-reduction parameters (e.g. normalization, background, sample thickness) are included. Several options are available for both resolution and Doppler broadening, including a crystal-lattice model for Doppler broadening. Self-shielding and multiple-scattering correction options are available for analysis of capture cross sections.

**Figure 8:**
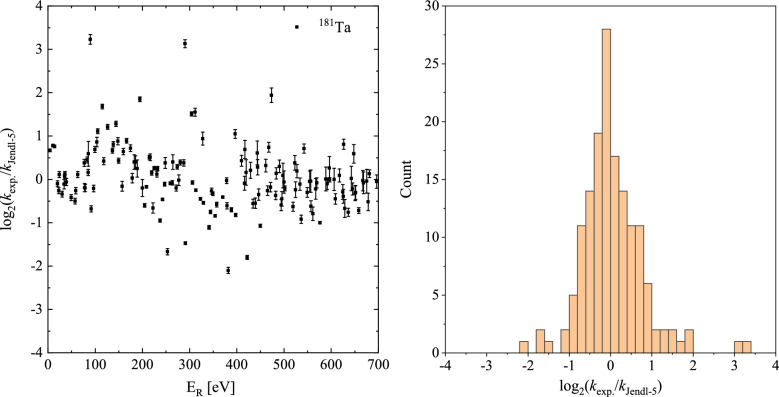
(**a**) The capture kernel *k* ratio $k_{exp}/k_\mathrm{JENDL-5}$ in 1–700 eV range and (**b**) its distribution.

Bayesian fitting of R-matrix resonance parameters implemented in SAMMY combines prior resonance parameters values and uncertainties with measured data and data uncertainties to yield updated parameter values and uncertainties. The SAMMY program analyzes the resonance parameters in three main steps: First, the initial values of the resonance parameters are used to calculate the theoretical values through the multienergy R-matrix theoretical cross-section formula; Second, various experimental conditions are simulated using the theoretical model or formula, such as: Doppler broadening, resolution, multiple-scattering, and the effect of self-shielding on the measurement results; Third, the experimental data are fitted using the Bayesian method analysis to obtain the new resonance parameters.

However, some individual resonance parameters,such as resonance spin J and partial neutron and radiative widths $\Gamma _{n}$ and $\Gamma _{\gamma }$ could not be determined reliably by experimental capture data. In general, only energy and capture kernel k, defined as

15 $$\begin{aligned} k =g \frac{\Gamma _{n}\Gamma _{\gamma }}{\Gamma _{n}+\Gamma _{\gamma }}, \end{aligned}$$

can be obtained reliably. The statistical factor g is given by

16 $$\begin{aligned} g =\frac{2J+1}{(2s+1)(2I+1)}, \end{aligned}$$

where *J* is the resonance spin, the neutron spin $s = 1/2$, and the ground state spin of the target nucleus $I(^{181}Ta) = 3.5^{+}$,hence in our case $g = (2J + 1)/16$. Resonance structures could be resolved up to neutron energies of 700 eV. For energy above 700 eV, the analysis of individual resonance parameters became increasingly difficult, due to the worsening of the experimental resolution at Back-n and the lower counting statistics. The capture kernel k extracted from experimental data are close for most resonance energies of JENDL-5, as shown in Fig. 8. For comparison, the logarithmic ratios of the kernels obtained from this work and from JENDL-5 are listed in the Supplementary Information of this work.

**Figure 9:**
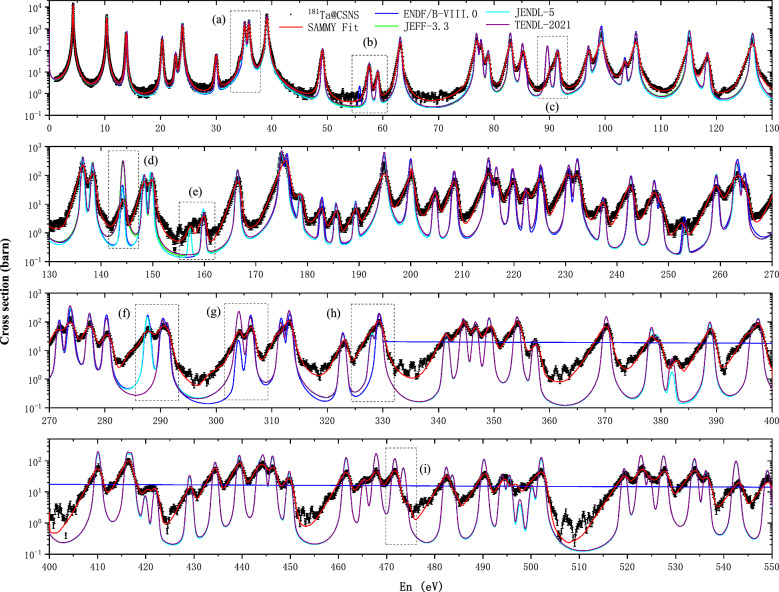
Comparison of $^{181}$Ta$(n,\gamma )$ cross sections with JENDL-5, TENDL-2019,JEFF3.3 and ENDF/B VIII.0 library 1–500 eV.

### Comparison with libraries

The final SAMMY fitted results of tantalum target are shown in Fig. 9. The black data represent the tantalum capture cross section measured in this work, and the red solid curve is the SAMMY fitted values to the present data. The fitted resonance energy $E_R$ and the radiative kernels derived using Eq. (15) are listed in the Supplementary Information of this work, and kernels calculated from JENDL-5 are still listed. From Fig. 9, it can be seen that in the 1–700 eV region, the new data show a good agreement with libraries in general. With respect to the ENDF/B-VIII, JENDL-5, TENDL-2021 and JEFF3.3 libraries, we observe

(a) the resonance at 34 eV not included in the ENDF/B VIII library, our experiments data showed support for JENDL-5, TENDL-2019 and JEFF3.3.

(b) the resonance at 56 eV included in the ENDF/B VIII.0 library, which is not confirmed by our experiments data.

(c) the cross section and $\hbox {E}_n$ at 90eV resonance has a clear difference between our experiments data and the libraries.

(d) the resonance at 143 eV, our experiments data support for the cross section of ENDF/B-VIII and JENDL-5.

(e) the resonance at 157 eV, our experiments data support for the cross section of JENDL-5 and JEFF3.3.

(f) the resonance at 287 eV not included in the TENDL-2019 library, our experiments data showed support for JENDL-5, ENDF/B VIII and JEFF3.3.

(g) the resonance at 303 eV, our cross section lay between ENDF/B-VIII and other three libraries.

(h) the resonance at 327 eV not included in the ENDF/B VIII library, our experiments data showed support for TENDL-2021, JENDL-5, and JEFF3.3.

(i) the resonance at 473 eV included in the JENDL-5, TENDL-2021 and JEFF3.3 library, which is not confirmed by our experiments data.

In the above comparison in the resolved resonance region below 700 eV, we can find that the experimental data are in good agreement with the JENDL-5 data, but there are also different regions (such as region near 90 eV and 473 eV). After investigation, it was found that the resonance parameters of JENDL-5 mainly refer to the work reported by Tsubone et al.^40^ in 1987, and its resolution is low below 510 eV. For the differences in resonance parameters between JENDL-5 and the present experiments, higher precision measurements are also required for cross-validation.

**Figure 10:**
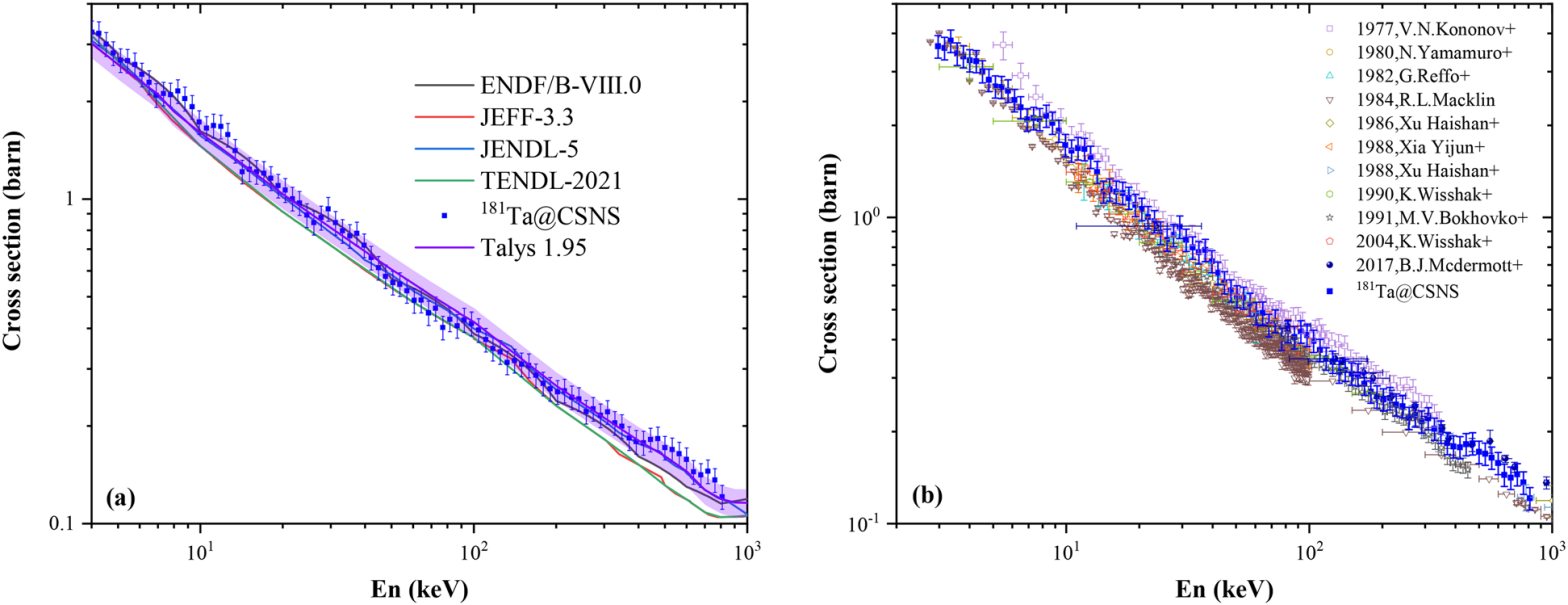
(**a**) Comparison of $^{181}$Ta$(n,\gamma )$ cross sections with four evaluated libraries; (**b**) Comparison of $^{181}$Ta$(n,\gamma )$ cross sections with existing experimental data from EXFOR library.

In Fig. 10, the averaged cross sections obtained in this work in the unresolved resonance region are compared with previous experimental results and the evaluated database. Figure 10a shows the comparison between our data and ENDF/B-VIII.0, JENDL-5, JEFF-3.3 and TENDL-2021 libraries. We can find that the experimental data are consistent with ENDF/B-VIII.0 (4–100 keV) and JENDL-5 (50–800 keV) in most areas, which are both generally higher than JEFF3.3 and TENDL-2021.

The TALYS 1.95 was used to describe the average cross sections in the URR. The calculations were based on the Hauser–Feshbach statistical emission model, which assumes that the capture reactions occur by means of a compound nuclear system that reaches a statistical equilibrium. The obtained statistical average level space $D_0$ average radiation width $<\Gamma _\gamma>$ in the resolved resonance region were used as input parameters for the TALYS code calculations. In addition, the global neutron optical model potential of Ref.^41^ was used in the calculations and other parameters are chosen with method reported in Chen et al.^42^, photon strength function is given by Kopecky and Uhl^43^, level density *a* and nuclear temperature *T* are given by Gilbert-Cameron model with adjusted parameters. The calculated capture cross sections well reproduced the experimental average cross sections of ${}^{181}$Ta as illustrated in Fig. 10(a).

Then, we compare the new data with the existing experimental data in unresolved resonance region, as shown in Fig. 10b. It shows that the new data are in agreement with the experimental results by Moxon et al.^44^, Yamamuro et al.^45^ and Macklin et al.^46^ in 2.4–10 keV, Kononov^47^ in 10–100 keV, Lindner et al.^48^ and McDermott^8^ in 0.1–1 MeV.

### Maxwell average cross section

For further applications of the (n,$\gamma$) cross section in the study of s-processes, the experimentally measured relevant data must be convolved with the neutron velocity distribution in the stellar plasma to obtain the Maxwellian-averaged cross section. The calculation of MACS requires a capture cross section over a sufficiently wide range of neutron energies, ideally from about 100 eV to 500 keV. This would be sufficient to cover the entire temperature range of the s-process scenario, including the highest temperatures reached during carbon shell burning in massive stars. According to the definition of the MACS^49^:

17 $$\begin{aligned} \begin{aligned} \text {MACS}(kT)&=\frac{\langle \sigma \vartheta \rangle }{\vartheta _T}\\&=\frac{2}{\sqrt{\pi }} \frac{\int _{0}^{\infty } \sigma \left( E_n\right) E_n e^{-\frac{E_n}{k T}} d E_n}{(k T)^{2}}, \end{aligned} \end{aligned}$$

where $\vartheta _T$ is the thermal velocity, *kT* = 30 keV is the characteristic thermal energy of an astrophysical site^50^. In this work, we mesured the MACS of $^{181}$Ta$(n,\gamma )$ at *kT* = 30 keV equal to 834 ± 75mb.

**Figure 11:**
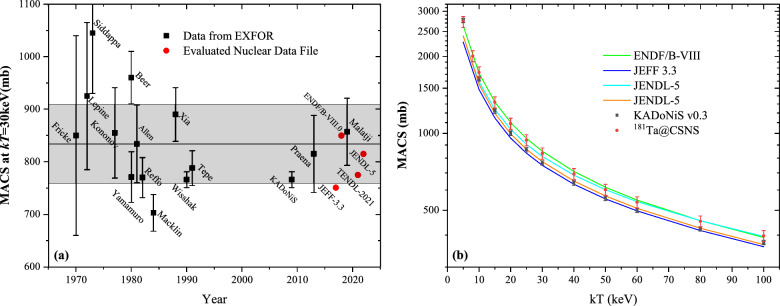
(**a**) MACS of $^{181}$Ta$(n,\gamma )$ at *kT* = 30 keV; (**b**) MACS of $^{181}$Ta$(n,\gamma )$ from *kT* = 5 to 100 keV, obtained in this work in comparison with evaluated databases and recommended values compiled in KADoNiS.

Fig.11a exhibit the MACS at *kT* = 30 keV obtained in the present work in comparison with evaluated nuclear data and existing experimental data, including Karlsruhe Astrophysical library of Nucleosynthesis in Stars(KADoNiS). It can be seen that most of the data were between 700 and 1050 mb, so is our data. Moreover, the experimental data is very close to latest experimental MACS obtained by Praena et al.^7^ and Malatji et al.^6^ and MACS derived from ENDF/B-VIII.0 and JENDL-5, as shown in Fig.11a. In particular, the present MACS shows an obvious discrepancy with KADoNiS recommended value 766 ± 15 mb.

In current celestial models, low-mass Asymptotic Giants Branch (AGB) star undergo s-processes at lower temperatures, while large-mass AGB star undergo s-processes at higher temperatures, ranging from 5 keV to 100 keV in the model. Therefore the MACS in this range is also an important parameter. Table 3 lists MACS values determined in this work from *kT* = 5 to 100 keV. MACS in this work are compared to recommended values in the Karlsruhe Astrophysical Database of Nucleosynthesis in Stars (KADoNiS)^51^ and values derived from JENDL-5^12^. Comparison our results with evaluated databases and recommended values compiled in KADoNiS are illustrated in Fig. 11). MACS for ${}^{181}$Ta of this work basically located between database JENDL-5 and ENDF/B-VIII. And our calculate values is obvoiusly higher than KADoNiS’s recommended values in general.

**Table 3:** MACS of $$ ^{181} $$Ta$$ (n,\gamma ) $$ for thermal energies 5$$< kT < $$100 keV.

*kT* (keV)	MACS (mb)		
	KADoNiS	JENDL-5	This work
5	2765	2714	2725 ± 245
10	1633	1665	1730 ± 156
15	1221	1267	1321 ± 119
20	1002	1051	1090 ± 98
25	862	912	940 ± 85
30	766	815	834 ± 75
40	639	685	693 ± 62
50	557	599	602 ± 54
60	500	538	537 ± 48
70		491	489 ± 44
80	423	454	452 ± 41
90		424	422 ± 38
100	374	398	397 ± 36

## Summary and conclusions

The $^{181}$Ta$(n,\gamma )$ cross section has been measured at the neutron time-of-flight facility Back-n of CSNS by using the four C$_{6}$D$_{6}$ liquid scintillator detectors. The experimental platform as well as the detector characteristics are briefly described, and the data analysis method is highlighted. The resonance parameters extracted from experimental data are given and analyzed using the R-matrix code in the resolved resonance region. Data in the unresolved resonance region are reported, which shows a good agreement with the JENDL-5 and ENDF/B-VIII.0, with some significant exceptions for small resonances. This work also gives the MACS from *kT*=5 to 100 keV over a sufficiently wide range of neutron energies, especially a value of 834 ± 75 mb at *kT* = 30 keV, which provides important reference data for *s*-process and stellar evolution in astrophysics. The new measurements strongly constrain the MACS of $^{181}$Ta($n,\gamma$) reaction in the stellar s-process temperatures.

### Supplementary Information

Supplementary Information.

## Data Availability

The $^{181}$Ta samples were measured for 17 h at a proton power of 125 kW. Then, $^{nat}$C and $^{nat}$Pb and empty target holder were measured for 8, 10 and 13 h respectively, accumulating more than 2 TB of data. These data that support the findings of this study are available from the corresponding author, [Zhendong An, anzhendong@mail.sysu.edu.cn], upon reasonable request.
